# Overcoming genetic neuromuscular diagnostic pitfalls in a middle-income country

**DOI:** 10.1093/braincomms/fcae342

**Published:** 2024-11-14

**Authors:** Rodrigo Siqueira Soares Frezatti, Pedro José Tomaselli, Christopher J Record, Lindsay A Wilson, Gustavo Maximiano Alves, Natalia Dominik, Stephanie Efthymiou, Krutik Patel, Jana Vandrovcova, Roope Männikkö, Robert D S Pitceathly, Claudia Ferreira da Rosa Sobreira, Robert McFarland, Robert W Taylor, Henry Houlden, Michael G Hanna, Mary M Reilly, Wilson Marques

**Affiliations:** Department of Neurosciences and Behaviour Sciences, Neuromuscular Disorders, University of São Paulo, Ribeirao Preto 14040-900, Brazil; Department of Neurosciences and Behaviour Sciences, Neuromuscular Disorders, University of São Paulo, Ribeirao Preto 14040-900, Brazil; Department of Neuromuscular Diseases, Queen Square Centre for Neuromuscular Diseases, UCL Queen Square Institute of Neurology, London WC1N 3BG, UK; Department of Neuromuscular Diseases, Queen Square Centre for Neuromuscular Diseases, UCL Queen Square Institute of Neurology, London WC1N 3BG, UK; Department of Neurosciences and Behaviour Sciences, Neuromuscular Disorders, University of São Paulo, Ribeirao Preto 14040-900, Brazil; Department of Neuromuscular Diseases, Queen Square Centre for Neuromuscular Diseases, UCL Queen Square Institute of Neurology, London WC1N 3BG, UK; Department of Neuromuscular Diseases, Queen Square Centre for Neuromuscular Diseases, UCL Queen Square Institute of Neurology, London WC1N 3BG, UK; Faculty of Medical Sciences, Translational and Clinical Research Institute, Newcastle University, Newcastle upon Tyne NE2 4HH, UK; Department of Neuromuscular Diseases, Queen Square Centre for Neuromuscular Diseases, UCL Queen Square Institute of Neurology, London WC1N 3BG, UK; Department of Neuromuscular Diseases, Queen Square Centre for Neuromuscular Diseases, UCL Queen Square Institute of Neurology, London WC1N 3BG, UK; Department of Neuromuscular Diseases, Queen Square Centre for Neuromuscular Diseases, UCL Queen Square Institute of Neurology, London WC1N 3BG, UK; NHS Highly Specialised Service for Rare Mitochondrial Disorders, Queen Square Centre for Neuromuscular Diseases, The National Hospital for Neurology and Neurosurgery, London WC1N 3BG, UK; Department of Neurosciences and Behaviour Sciences, Neuromuscular Disorders, University of São Paulo, Ribeirao Preto 14040-900, Brazil; Translational and Clinical Research Institute, Faculty of Medical Sciences, Wellcome Centre for Mitochondrial Research, Newcastle University, Newcastle upon Tyne NE2 4HH, UK; NHS Highly Specialised Service for Rare Mitochondrial Disorders, Newcastle upon Tyne Hospitals NHS Foundation Trust, Newcastle upon Tyne NE1 4LP, UK; Translational and Clinical Research Institute, Faculty of Medical Sciences, Wellcome Centre for Mitochondrial Research, Newcastle University, Newcastle upon Tyne NE2 4HH, UK; NHS Highly Specialised Service for Rare Mitochondrial Disorders, Newcastle upon Tyne Hospitals NHS Foundation Trust, Newcastle upon Tyne NE1 4LP, UK; Department of Neuromuscular Diseases, Queen Square Centre for Neuromuscular Diseases, UCL Queen Square Institute of Neurology, London WC1N 3BG, UK; Department of Neuromuscular Diseases, Queen Square Centre for Neuromuscular Diseases, UCL Queen Square Institute of Neurology, London WC1N 3BG, UK; NHS Highly Specialised Service for Rare Mitochondrial Disorders, Queen Square Centre for Neuromuscular Diseases, The National Hospital for Neurology and Neurosurgery, London WC1N 3BG, UK; Department of Neuromuscular Diseases, Queen Square Centre for Neuromuscular Diseases, UCL Queen Square Institute of Neurology, London WC1N 3BG, UK; Department of Neurosciences and Behaviour Sciences, Neuromuscular Disorders, University of São Paulo, Ribeirao Preto 14040-900, Brazil; National Institute of Sciences and Technology (INCT)-Translational Medicine Conselho Nacional de Desenvolvimento Científico e Tecnológico (CNPq) e Fundo de Amparo à Pesquisa do Estado de São Paulo (FAPESP), Ribeirao Preto, São Paulo 14040-900, Brazil

**Keywords:** genomic medicine, diagnostic pitfalls, capacity building, middle income country, transcontinental consortium

## Abstract

Neuromuscular disorders affect almost 20 million people worldwide. Advances in molecular diagnosis have provided valuable insights into neuromuscular disorders, allowing for improved standards of care and targeted therapeutic approaches. Despite this progress, access to genomic diagnosis remains scarce and inconsistent in middle-income countries such as Brazil. The lack of public health policies to enable feasible genetic diagnosis and the shortage of neuromuscular disorders specialists are the main reasons in this process. We report our experience in a transcontinental genomic consortium for neuromuscular disorders highlighting how collaborative efforts have helped overcome various obstacles in diagnosing our patients. We describe several challenging cases categorized into three major themes, underlining significant gaps in genetic diagnosis: (i) reverse phenotyping and variant validation, (ii) deep phenotyping and identifying a bespoke molecular approach, and (iii) exploring the use of genomic tests beyond whole exome sequencing. We applied a qualitative case-based approach to exemplify common pitfalls in genomic diagnosis in a middle-income country. Our experience has shown that establishing a virtual transcontinental partnership is viable, offering effective exchange of scientific experiences, providing both guidance for rational decision-making and specialized training on a local level and access to diverse molecular diagnosis strategies and functional analyses. Collaborative efforts such as these have the potential to overcome local obstacles, strengthen scientific capabilities, foster diverse multi-ethnic cohorts, and ultimately provide improved care for patients.

## Teaser

Frezatti et al applied a qualitative case-based approach to exemplify common pitfalls in genomic diagnosis in a middle-income country. They report how establishing a virtual transcontinental partnership is viable, and can help to overcome local obstacles, strengthen scientific capabilities, avoid misdiagnosis, and ultimately provide improved care for patients.

## Graphical Abstract

**Graphical Abstract:**
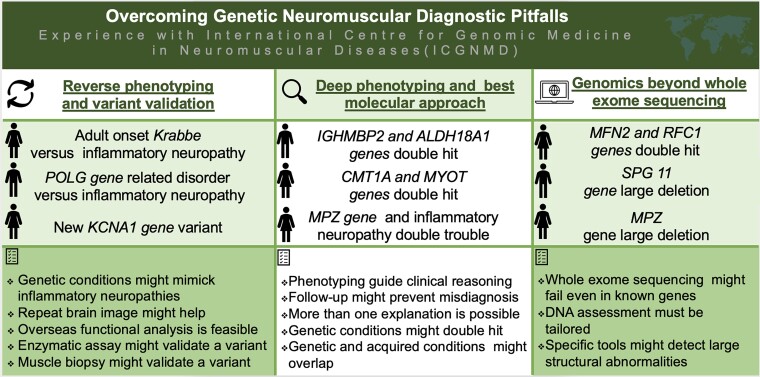

## Introduction

Neuromuscular disorders (NMDs) encompass a wide group of conditions that can be broadly classified as acquired or inherited.^1^ The peripheral nervous system is an important component and may be affected in its sensory and/or motor pathways. Neuromuscular motor dysfunction may arise from diseases affecting motor neurons including the anterior horn cells, motor axons, ion channels and diseases of the neuromuscular junction and the muscle.^2^ From a sensory perspective, dysfunction can arise from diseases affecting the dorsal root ganglion and/or the sensory axons. The cranial nerves can also be affected in NMDs. Some NMDs are defined as complex and include involvement outside the neuromuscular system including the central nervous system and even non-neurological systems, highlighting various possible disease mechanisms, and underpinning the complexity of DNA-based diagnosis.^3^ Inherited NMD are mainly monogenic and can display autosomal recessive, autosomal dominant, X-linked and mitochondrial inheritance. Collectively, prevalence ranges from 10 to 100 per 100.000 individuals, affecting nearly 20 million people worldwide.^4^ Advances in molecular diagnosis have provided valuable insights into this complex group of disorders, allowing for improved standards of care and targeted therapeutic approaches. Comprehensive genetic testing has become a reality in high-income countries, where it is available in a reasonably affordable manner with rapid turnaround times. Furthermore, it is gradually being integrated into clinical practice in low and middle-income countries, although progress there is rather more constrained and erratic than in high income countries and hampered by several difficulties with implementation.^5^ In such countries, Brazil included, comprehensive molecular testing is not feasible in a uniform manner. Moreover, accurate selection of best genetic test and result interpretation is challenging and depends on clinicians being adequately trained and equipped to do so. Massive genetic data acquisition is running much faster than the availability of clinicians with expertize to interpret it.

To tackle these problems, a multi-centre translational collaboration is a welcome strategy that can provide: exchange of scientific experiences, specialized training on a local level and access to diverse molecular diagnostic strategies and functional analysis. Collaborative efforts such as these have the potential to overcome local obstacles, strengthen scientific capabilities, foster diverse multi-ethnic cohorts and ultimately provide improved care for patients.

Herein, we describe our experience with the International Consortium for Genomics in Neuromuscular Disorders (ICGNMD)^6^ illustrated by challenging clinical cases, grouped into three major categories: (i) reverse phenotyping and variant validation, (ii) deep phenotyping and identifying the best molecular approach and (iii) exploring the utility of genomic tests beyond whole exome sequencing (WES). The clinical reasoning behind the diagnosis of these cases highlights common pitfalls in neurogenetic diagnosis and how a transcontinental consortium has helped to overcome these challenges.

## Materials and methods

The ICGNMD has 18 NMD centres in seven different countries (Brazil, India, Netherlands, South Africa, Turkey, UK and Zambia) headed by UCL Queen Square Institute of Neurology. The consortium was launched in June 2019 and through a structured locally approved protocol aims to recruit NMD patients and relatives, sharing materials and data with a multi-specialist panel for the final study of a multi-ethnic cohort of NMD patients.

**Figure 1:**
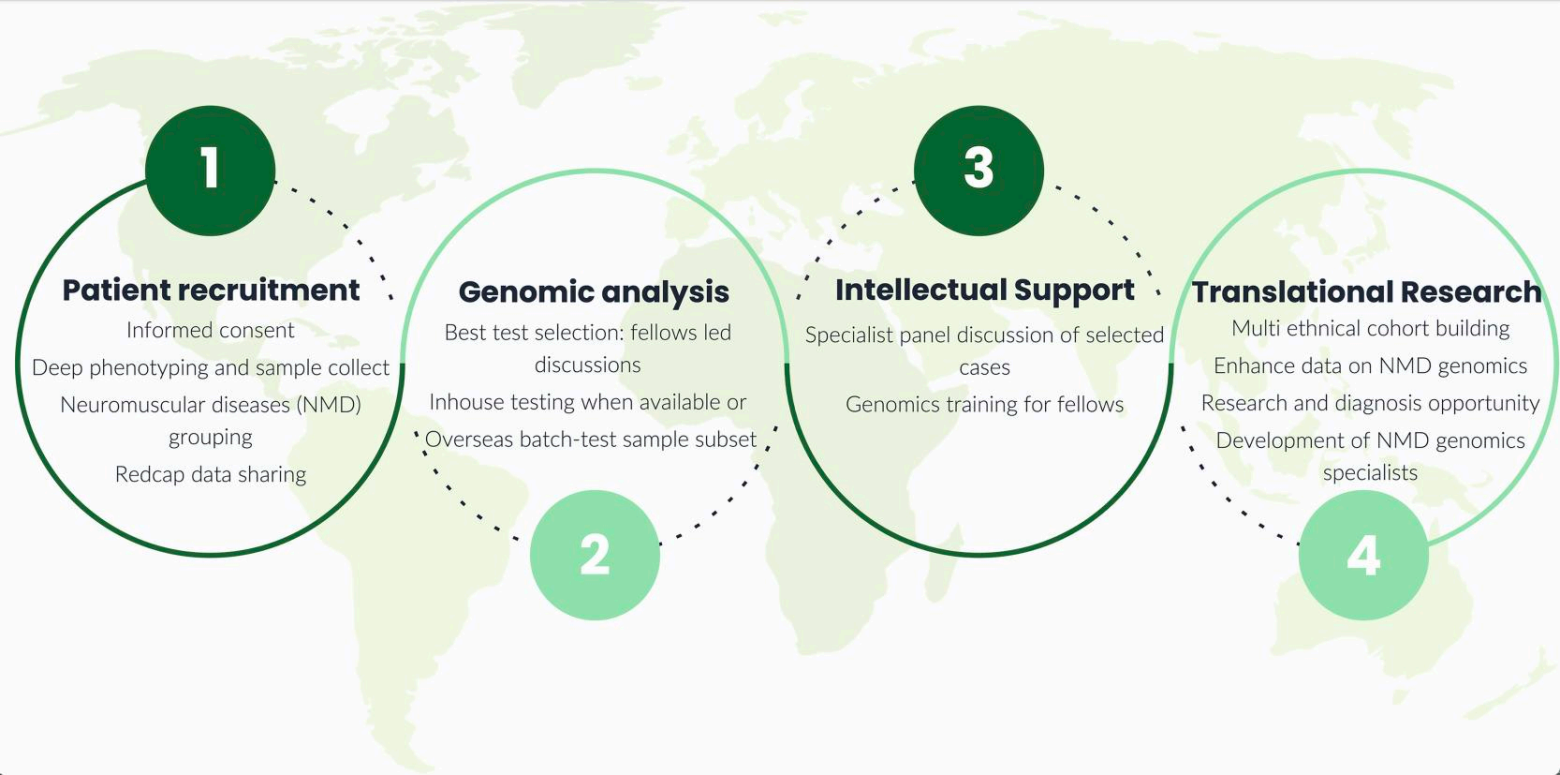
Steps involved in patient ´s inclusion on ICGNMD cohort.

Clinical data are systematically gathered and uploaded to a bespoke Redcap (Research Electronic Data Capture) database using HPO terms, ensuring anonymity of the participants. Informed consent was diligently obtained from all patients or their authorized representatives. In Brazil, initial genetic testing was primarily conducted locally according to clinical suspicion, encompassing comprehensive sequencing of PMP22, GJB1, MPZ, GDAP1 and VAPB genes, along with evaluating chr17 dosage and PCR to detect repeat expansions in C9orf72, FTXN, SCA 1,2,3,6,7,8,10,12,17, DRLPA and RFC1. In cases where patients received negative test results or when the appropriate test was not available, an aliquot of their DNA was sent to UCL for further genetic analysis. The genomic analysis commenced by identifying the most suitable initial test, considering the clinical phenotype, familial relationships, results and tests already performed and the availability of DNA samples from relatives before either whole exome or whole genome sequencing were applied. This bespoke approach was facilitated by regular multi-disciplinary meetings between the UCL and Brazilian teams. Regular training sessions were conducted to create a translational research environment, enhance knowledge of local NMD prevalence and ensure ongoing education in neurogenetics for NMD (Fig. 1).

In the current study, we present a series of complex cases that were successfully solved through the collaborative network facilitated by the ICGNMD consortium. By emphasising certain pitfalls in genetic testing and underscoring, the significance of personalized interpretation of results, we demonstrate the crucial role of collaboration in achieving accurate diagnoses and optimal patient care.

We have grouped the cases into three main categories with the aim of highlighting what, in our experience, were the main obstacles in the diagnostic process. The strategies used in this process are then used in a continuum, applicable to other cases in our practice.

## Results

### Reverse phenotyping and variant validation

#### Clinical vignette 1

**Figure 2:**
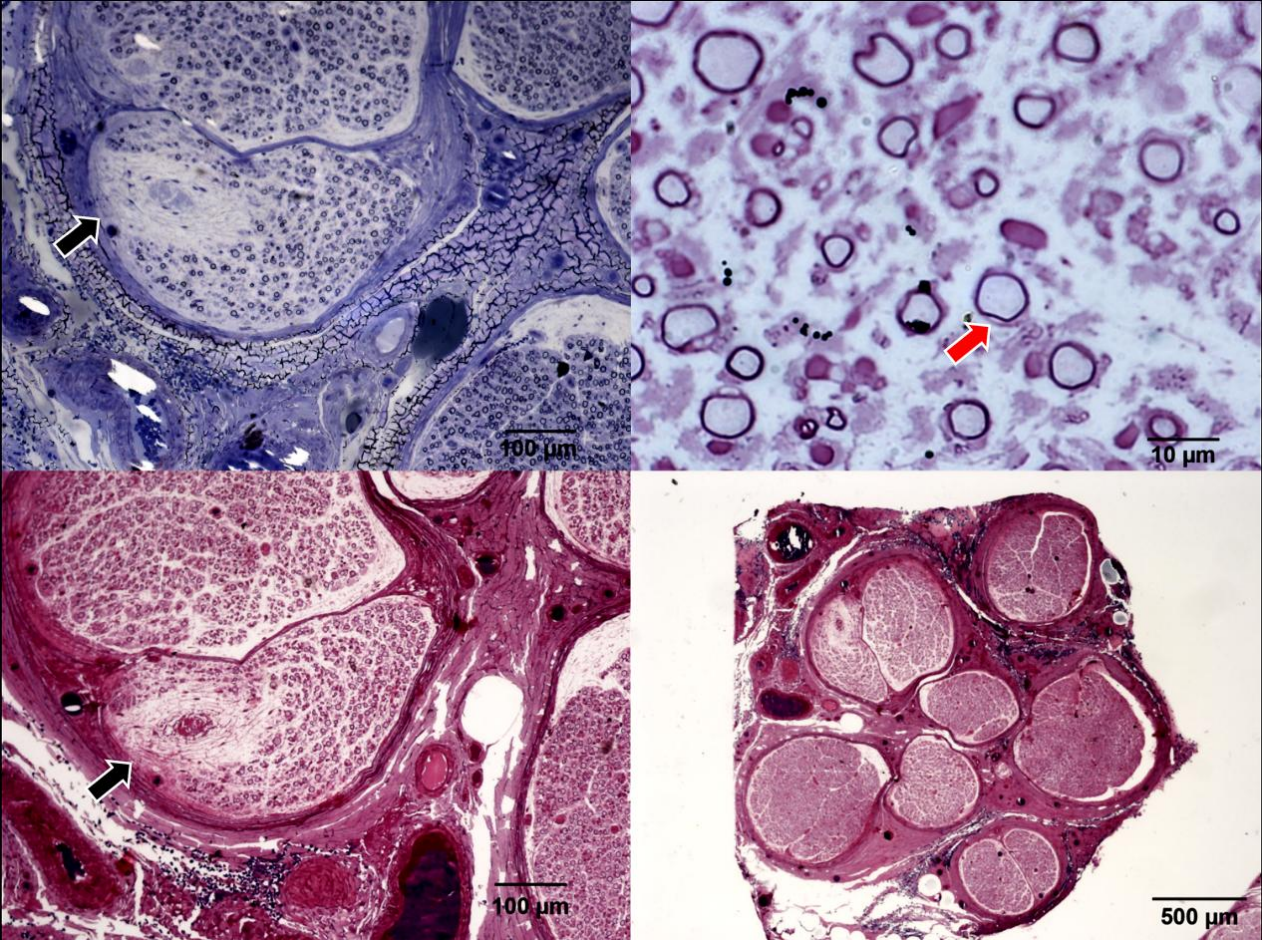
**Nerve biopsy of patient presented in clinical vignette 1.** Staining with toluidine blue in upper row and H&E in lower revealing reduction in the myelinated fibre population with overall thin myelin sheath for the fibre diameter (red arrow). Additionally, there are unspecified inclusions in cytoplasm and no inflammation (black arrows).

A 46-year-old woman, from healthy and non-consanguineous parents, presented at the age of 30 years with slowly progressive, symmetric, global weakness with upper limb, distal predominance associated with dysphonia and dysphagia. She had no cognitive impairment, no upper motor neuron signs, no cranial nerve involvement, and only distal sensory complaints in upper and lower limbs. Nerve conduction studies revealed decreased motor nerve conduction velocities within demyelinating range (ulnar amplitude = 8.8 mV/and CV = 31 m/s) and decreased sensory nerves action potentials amplitudes and conduction velocities. A brain MRI done at age of 37 years was normal. The available local tests were normal, including PMP22 dosage, PMP22 and GDAP1 sequencing. Brachial plexus MRI revealed symmetric intense thickening and post gadolinium enhancement. CSF revealed slightly elevated protein levels (40 mg/dL; reference range 8–32 mg/dL) with normal cell count (1 cell). A sural nerve biopsy revealed a significant reduction in the myelinated fibre population with overall thin myelin sheaths for the fibre diameter. Additionally, there were some unspecified inclusions in cytoplasm and no inflammation (Fig. 2). Considering the clinical features, neurophysiology and brachial plexus MRI we considered the diagnosis of CIDP. Patient was then treated with high doses of intravenous methylprednisolone and IVIG, with no response. Due to the poor response to treatment and the ongoing progression of symptoms, a genetic diagnosis was considered. WES was undertaken and revealed a homozygous class 3 variant (NM_000153.4:c.973A>G; p.Met325Val) in the *GALC* gene, raising the possibility of a rare form of adult onset Krabbe’s disease. Even though isolated demyelinating neuropathy had already been described previously in adult onset Krabbe’s, an upper limb predominance with vocal cord paresis was not reported.^7^ The variant is conserved (phyloP100: 5.086), absent in homozygous state in large database, including gnomAD and multiple lines of *in silico* prediction tools supports deleterious effect (Revel score 0.757). Nevertheless, to validate the variant we pursued enzymatic assay that revealed low levels of galactocerebrosidase in the serum, 4,8 nmol/17 h/mg protein (NR: 14–83 nmol/17 h/mg protein) confirming the diagnosis of Krabbe’s disease. Repeated brain MRI revealed minor white matter hyperintensities along occipital horns (Supplementary Fig. 1).

#### Clinical vignette 2

An 8-year-old boy, from healthy and non-consanguineous parents (Supplementary Fig. 2) presented at the age of 3 years with a progressive loss of manual dexterity, particularly difficulty in grasping and manipulating small objects. He had no cognitive impairment or other neurological symptoms. On neurological examination there was mild symmetrical proximal weakness in the lower limbs and no ataxia or ophthalmoplegia. Power was diminished distally and symmetrically in both upper limbs. NCS showed a symmetric sensory-motor neuropathy with intermediate conduction velocity slowing (ulnar 3 mV amplitude and 43 m/s CV). Laboratory tests revealed increased serum lactate, 6,9 mmol/L (NR 05 −2 mmol/L), and elevated protein levels in CSF, 417 mg/dL (NR 15–60 mg/dL). The diagnosis of CIDP was considered and IVIG treatment commenced, however with no effect. Molecular diagnosis was then pursued, and WES revealed two heterozygous variants in the *POLG* gene (NM_002693.3:c.2557C>T;p.Arg853Trp and NM_002693.3:c.1943C>T; p.Pro648Arg). Segregation analysis confirmed the variants were in *trans*. The first variant is ACMG class 5, absent in homozygosity in large database, including gnomAD and multiple lines of *in silico* prediction tools supports deleterious effect (Revel score 0.881). Second variant is ACMG class 4, highly conserved (phyloP100: 7.823), absent in large database, including gnomAD and multiple lines of *in silico* prediction supports deleterious effect (Revel score 0.941).

**Figure 3:**
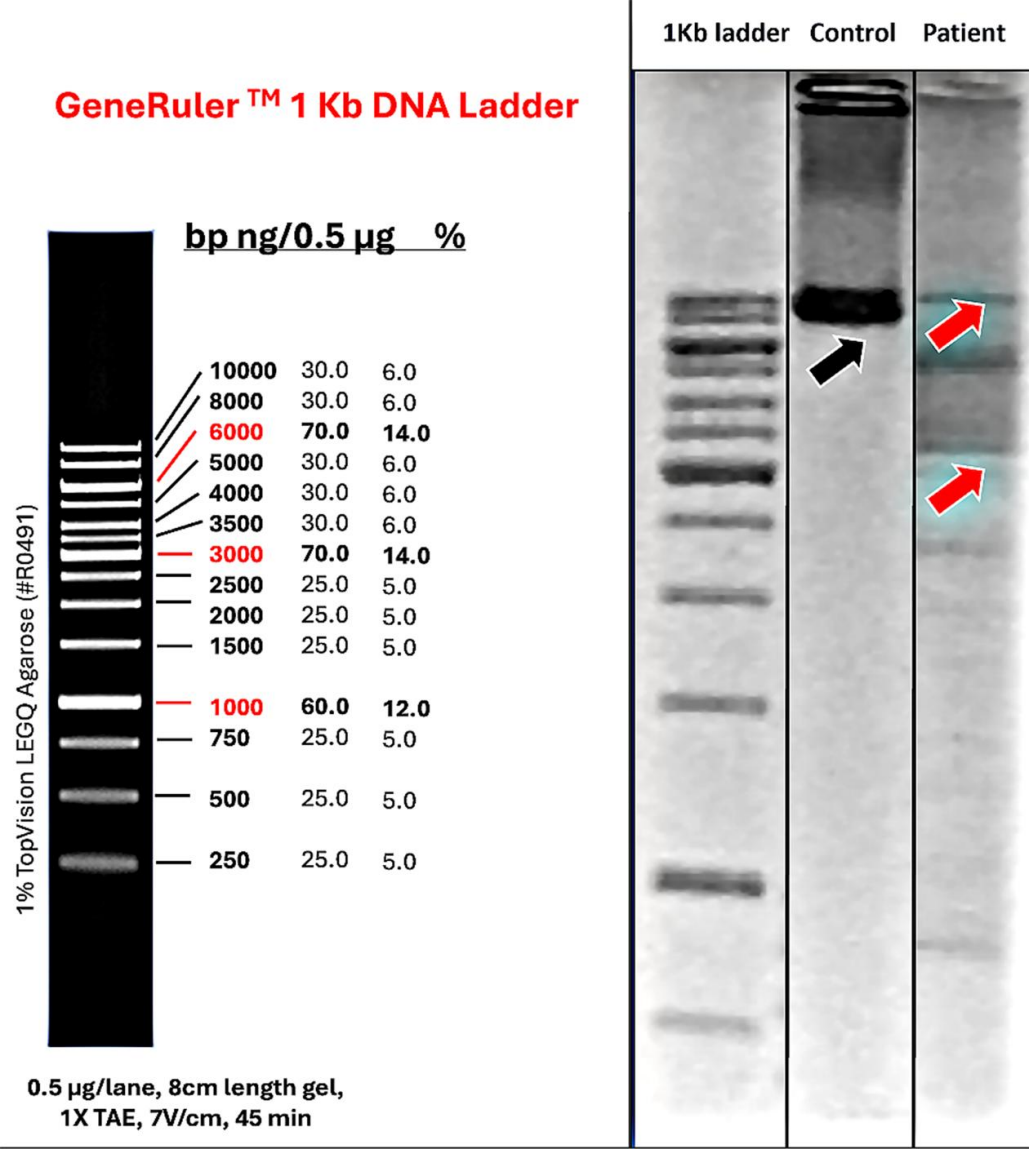
**PCR 1% agarose gel electrophoresis**. Showing mitochondrial DNA multi-deletion of patient depicted in clinical vignette 2. In the left, 1 Kb ladder and its size correlation that allows to detect shorter products (red arrows) in patient’s material compared with WT control (black arrow) indicating the presence of a mtDNA deletion. The figure was cropped to optimize description. Original, uncropped gel is provided in Supplementary Fig. 4.

**Figure 4:**
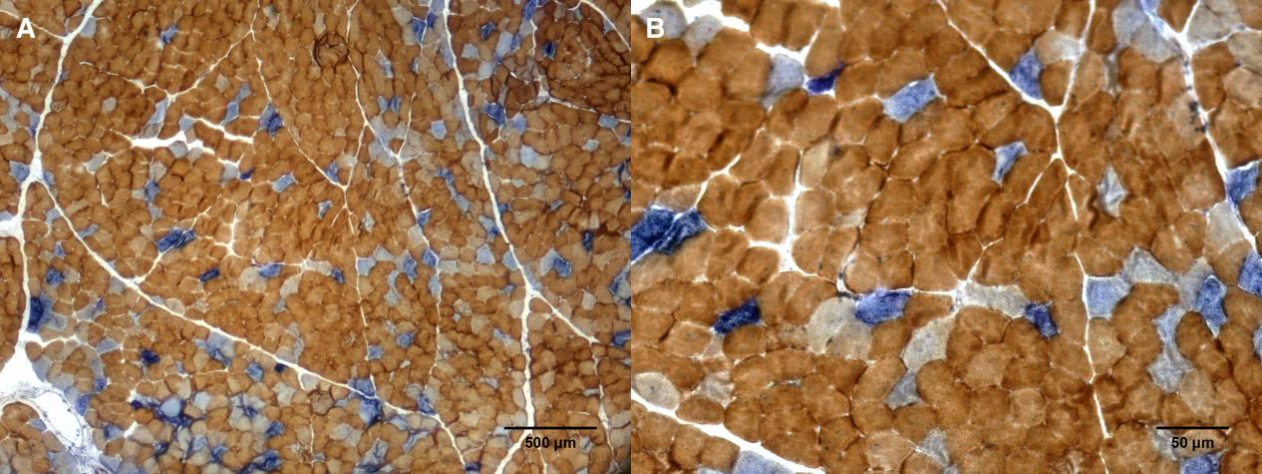
**Muscle biopsy of patient presented in clinical vignette 2**. Stained with COX-SDH in 500 μm zoom in Panel (**A**) and 50 μm zoom in Panel (**B**). Blue stain from SDH (complex II that depends solely on nDNA) overpasses brown stain from COX negative fibres (complex IV, which depends on mtDNA and nDNA). COX, cytochrome c oxidase; SDH, succinate dehydrogenase; nDNA, nuclear deoxyribonucleic acid; mtDNA, mitochondrial deoxyribonucleic acid.

Although some clinical features were consistent with those described with this genotype, there was no ophthalmoplegia or myopathic findings, typically associated with the condition. A muscle biopsy was then pursued and when stained with SDH-COX revealed COX negative fibres which along with multiple deletions of mitochondrial DNA, further supported the role of the *POLG* variants as the cause of the disease (Fig. 3 and Supplementary Fig. 1 with uncropped version and Fig. 4). Reverse phenotyping and functional analysis played a crucial role in reaching the final diagnosis.

#### Clinical vignette 3

**Figure 5:**
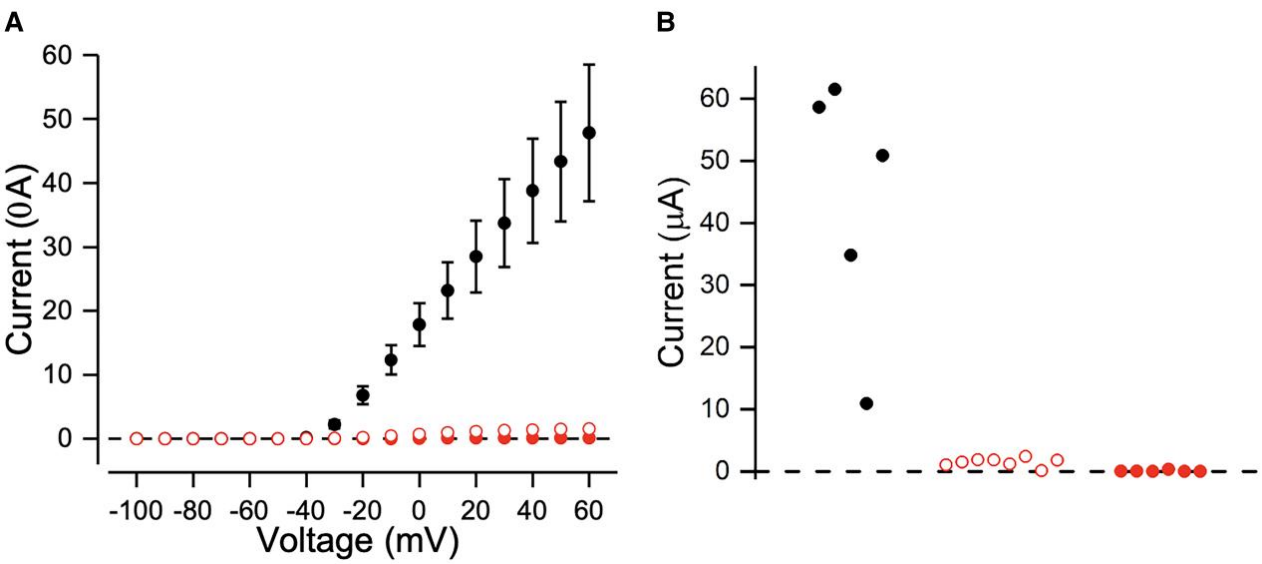
**Current—voltage relationship of patient presented in clinical vignette 3**. (**A**) WT, homomeric and simulated heterozygous R301K channels, simulating what has been found in patient depicted in clinical vignette 3. KCNA1 R301K mRNA was injected into *X. laevis* oocytes on its own or together with WT mRNA (simulated heterozygous condition) and the current features were compared to those injected with WT mRNA alone (*n* = 5, black). No currents were detected on oocytes injected with R301K (*n* = 6, red, solid symbols) mRNA suggesting full loss of function. When co-expressed with WT mRNA, the current amplitude was significantly suppressed compared to WT, suggesting a dominant negative effect of R301K subunit in WT subunits in the channel tetramer (*n* = 8, red, open symbols), pointing towards pathogenicity of the variant and thus explaining our patient´s phenotype. Holding voltage was −80 mV. Currents were measured at the end of test voltage pulses ranging from −100 to +60 mV in 10 mV increments. Dashed line shows zero-current level. Panel **B** shows current amplitude for individual cells in response to voltage step to +50 mV.

A 36-year-old woman presented with recurring headaches accompanied by brainstem aura, such as tinnitus and motor impairment. These symptoms began when she was 10 years old and later became associated with focal dysperceptive seizures. At the age of 20 year, she experienced progressive appendicular cerebellar ataxia and orbicularis oculi myokymia. Her father was also affected with the same phenotype, and her son had only migraine and ataxia. Brain MRI revealed cerebellar vermis atrophy. EEG showed right frontoparietal epileptic discharges. NCS were normal and needle examination revealed myokymic discharges in the orbicularis oculi muscles. Genetic testing for SCA 1,2,3,6,7,10 and common mitochondrial point mutations yielded negative results. WES revealed a class 3 heterozygous *KCNA1* variant (NM_000217.3: c.902G>A; p.Arg301Lys), which was absent in large database, including gnomAD, and for which *in silico* analyses predicted a deleterious effect (Revel: 0914). Sanger sequencing confirmed that the variant was present in both other affected family members (her father and her son) (Supplementary Fig. 3). Further functional analysis was conducted through the consortium suggesting a dominant negative effect of this variant. We used a Xenopus oocyte model to evaluate voltage relationship of wild type (WT), homomeric and simulated heterozygous R301K channels. KCNA1 R301K mRNA was injected into *Xenopus laevis* oocytes on its own or together with WT mRNA (simulated heterozygous condition) and the current features were compared to those injected with WT mRNA alone. No currents were detected on oocytes injected with R301K mRNA suggesting full loss of function. When co-expressed with WT mRNA, the current amplitude was significantly suppressed compared to WT, suggesting a dominant negative effect of R301K subunit in WT subunits in the channel tetramer (Fig. 5).

### Deep phenotyping and identification of the best molecular approach

#### Clinical vignette 4

A 14-year-old male patient, from consanguineous parents (Supplementary Fig. 5), presented at age of 1 year, with a slowly progressive, symmetrical, distal predominant weakness. He also experienced severe joint hypermobility and spinal deformities. NCS was normal and needle examination revealed diffuse chronic denervation. WES first revealed a homozygous class 5 variant in the *IGHMBP2* gene (NM_002180.3: c.2796delC, p.Cys932TrpfsTer46). It is a frameshift variant, absent in large database, including gnomAD related to distal hereditary motor neuropathy (dHMN) type VI and CMT2S. The alteration, however, failed to explain the hypermobility. Raw data reanalysis including hypermobility among the HPO terms, facilitated the discovery of a second class 4 homozygous variant in the *ALDH18A1* gene (NM_002860.4: c.121 C>T, p.Arg41Cys), which is highly conserved (pyloP100: 6.656), absent in homozygosity in large databases, including gnomaD, and associated with *cutis laxa*, and thus explaining the full clinical picture. The successful identification of these variants highlights the importance of thorough phenotyping before selecting the appropriate targets and conducting a clinical reasoning-based re-analysis of the NGS data.

#### Clinical vignette 5

A 34-year-old woman with a definite diagnosis of CMT1A presented for follow-up. Since childhood she had difficulty walking, a moderate scoliosis, and a cervical muscle hypertrophy. Family attributed that to CMT1A, because of muscle overload due to distal weakness. In the first appointment power exam revealed the following MRC: neck extension and flexion 3/5; arms adduction, abduction, flexion, extension 2/5 bilaterally; first dorsal interosseus, abductor policis brevis (APB), abductor digiti minimi (ADM), wrist flexion and extension 2/5 bilaterally. In the lower limbs, hip flexion 2/5 bilaterally, hip extension and leg flexion 1/5 bilaterally and lastly leg extension, plantar dorsiflexion and plantar flexion 2/5 bilaterally. An outside electromyography reported only a uniform demyelinating sensory motor neuropathy with absent sensory nerve action potentials throughout, and motor nerve conduction study (MNCS) in ulnar nerve was absent in ADM but when recorded in flexor carpi ulnaris revealed: 14.3 ms of distal latency/1,5 mV of amplitude/22.7 m/s of conduction velocity. MNCS in median nerve, recording in APB, revealed: 9.8 ms of distal latency/0.2 mV of amplitude/34 m/s of conduction velocity. As the degree of proximal muscular weakness was not in tune with CMT1A expected progression, we considered a superimposed condition. Even though CK and aldolase were within normal limits, a new electromyography with needle examination of proximal muscles revealed a myopathic pattern in sternocleidomastoids, deltoids, iliopsoas and vastus medialis (Supplementary Fig. 6). WES revealed a class 5 heterozygous variant in the *MYOT* gene (NM_006790.2: c.179C>T; p.Ser60Phe), which is highly conserved (phyloP100: 5.433), has a low frequency in large databases, including gnmoAD (0.0 000 157) and predicted pathogenic in some *in silico* analysis, BayesDel addAF (0.1912), confirming another double hit genetic disorder with a superimposed late onset myofibrillar myopathy related to MYOT gene on top of CMT1A. Since then, regular cardiac assessment was commenced, and patient did not show any abnormalities so far.

#### Clinical vignette 6

A 30-year-old female, from healthy and non-consanguineous parents, presented at age of 5 year with a slowly progressive length-dependent sensorimotor neuropathy. At 25 year, NCS revealed a sensorimotor demyelinating neuropathy, with upper limb motor conduction velocities below 10 m/s. Furthermore, all nerves studied showed temporal dispersion. The local CMT panel showed a class 5 heterozygous variant in the *MPZ* gene (NM_000530.8: c.292C>T; p.Arg98Cys), which has a low frequency in large database, including gnomAD (0,00002), multiple lines of *in silico* prediction tools supports deleterious effect (Revel score 0.772) and is well described in literature.^8^ The patient evolved slowly over the years, in keeping with a CMT1B diagnosis. Nevertheless, at age 35, after an alleged Zika virus infection, she developed an acute exacerbation, marked by proximal weakness and severe gait instability. NCS at this stage did not change much and revealed intense temporal dispersion with possible conduction block, similarly to previous study (Supplementary Fig. 8). Despite this, assuming a superimposed inflammatory polyradiculoneuropathy, she was treated with IVIG 2 g/Kg. She improved significantly, becoming able to walk without assistance, to keep stable with eyes closed and to hold her new baby with her arms, an action that she never could do before with her other three children. According to her own words: ‘I have never felt so good in all my life’. Within a few months, however, weakness relapsed. Considering the clinical deterioration, previous response to immunomodulation and nerve conduction studies revealing temporal dispersion, we considered an overlap with CIDP, and put her on IVIG 1 g/Kg/month, leading to sustained clinical improvement and stabilisation. She became able to breast-feed her baby without help whereas previously, she required significant assistance.

### Exploration of genomic tests beyond WES

#### Clinical vignette 7

A 35-year-old woman, from non-consanguineous and healthy parents, (Supplementary Fig. 7) presented with slowly progressive imbalance and sporadic falls for 1 year. She had a mixed ataxia with distal weakness in the lower limbs. Brain MRI showed a possible asymmetric hypertrophic olivary degeneration (HOD; Supplementary Fig. 9) and EMG a sensory-motor axonal neuropathy. An extensive workup ruled out concomitant neoplasia, paraneoplastic antibodies, rheumatologic or infectious disorders. CSF was normal. After a few appointments, we evaluated her brother who had a history of distal sensory complaints. He actually had a sensory motor axonal neuropathy that turned out to be related to *MFN2* class 4 heterozygous variant (NM_014874.4:c.2207A>T;p.Asn736Ile), which is highly conserved (phyloP100: 8.911), not found in large database, including gnomaD, and multiple lines of *in silico* prediction tools supports deleterious effect (Revel score 0.947).

The same variant, also present on the proband failed to explain the whole clinical picture. We decided to go for WES looking for abnormalities in ataxia genes that were not found. After revisiting the phenotype, a vectoelectronystagmography revealed bilateral vestibular arreflexia, which made us consider CANVAS. A *RFC1* repeat expansion was positive only in proband and revealed a homozygous expansion confirming the co-existence of two Mendelian genetic disorders.

#### Clinical vignette 8

A 30-year-old man, born from consanguineous and healthy parents, presented with slowly progressive walking difficulties since his first decade of life. He also had problems learning and intellectual disabilities. Brain MRI showed marked atrophy of the corpus callosum. On examination there was a spastic paraparesis with distal predominant weakness and sphincter involvement. EMG showed diffuse involvement of the lower motor neuron. WES revealed no variants of interest. Given the high clinical probability of complex SPG, including SPG11 and 15, CNV was investigated, using an in-house bioinformatic CNV pipeline which revealed a homozygous deletion of *SPG 11* (deletion coordinates chr15: 44 569 313–44 575 076). This deletion was detected by all tools within the pipeline (clinCNV, ExomeDepth, cn.mops).^9-11^

#### Clinical vignette 9

A 30-year-old man presented in the first decade of life with symmetrical distal weakness and hypoesthesia affecting mainly the lower limbs. His father also displayed the same phenotype. NCS revealed a sensory and motor polyneuropathy with intermediate reduction in conduction velocity. WES revealed no good candidate. Thus, another in house validated targeted script assessment for CNV and large structural abnormalities using VEP data were pursued, revealing a large heterozygous deletion (8.1 Mb, NC_000001.10: g.(? 160 739.302) (161.549.844_?) including the *MPZ* gene, confirming CMT1B. Parental samples, specifically the father’s DNA, were not available for segregation studies.

## Discussion

Traditionally, phenotyping an individual with a suspected inherited condition has been the usual approach for selection of a bespoke genomic test. The so-called deep phenotyping is instrumental to narrow down aetiological possibilities and helps to strengthen phenotype–genotype correlation. However, with the declining costs and improved accessibility of next-generation sequencing (NGS), some experts propose an alternative approach: initially exploring genomics to assess the hypothesis of correlation, followed by a targeted clinical evaluation, known as reverse phenotyping.^12^

The importance of revisiting phenotypes is known, especially in ultra rare disorders (prevalence <1 in 50.000 individuals). Thorough characterisation and comprehensive evaluation of individuals ensure accurate genotype–phenotype correlation, particularly in cases involving atypical clinical presentations. In these settings, the implementation of virtual panels utilising HPO terms effectively enhances the detection rate due to the larger gene pool encompassed by such panels. Nevertheless, identifying and confirming a potential novel candidate gene responsible for an unusual clinical presentation, while exciting, demands an additional level of certainty. Unfortunately, the availability of animal models and advanced functional studies remains limited in low- and middle-income countries, making the process even more challenging.

While traditional medical reasoning considers all symptoms collectively to arrive at a definitive diagnosis, this approach may not always hold true. In practice, some clinical scenarios are challenging due to possible pitfalls that might arise from incorrect phenotyping or even lack thereof, highlighting the importance of deep and reverse phenotyping taking together. Special consideration should be given to: (i) double hit genetic disorders, (ii) genetic disorders overlapping with acquired condition and (iii) inherited conditions with characteristics resembling acquired disorders. In these situations, suspicion should arise when the clinical presentation or disease progression deviates from what is anticipated based on existing natural history studies. Detecting such discrepancies is essential as it may suggest the presence of atypical or rare conditions that require further investigation and a tailored approach to diagnosis and treatment.

In our study, the first six cases explored these pitfalls. In the first two cases, major issues included misdiagnosis as an acquired condition and the need for variant validation in unusual clinical presentations after molecular analysis.

In Case 1, we initially considered an acquired condition based on symptoms of neuropathy with conduction velocity slowing, albumino–cytologic dissociation, and progressive weakness with proximal involvement. The lack of treatment response, slow symptom progression, and vocal cord involvement suggested an inherited condition. However, NGS did not immediately solved the case as the homozygous GALC variants founded needed further validation. While prior studies have documented cases of isolated demyelinating neuropathy, the atypical presentation characterized by upper limb predominant weakness and vocal cord paresis had raised uncertainty regarding its interpretation.^7^ Repeated brain at this stage did show white matter hyperintensities near occipital horns, suggesting that it might aid in the diagnosis of complex neuropathies. Nevertheless, it was not enough to validate the variant and serum galactocerebrosidase dosage was finally employed to aid in the diagnosis of a rare adult onset Krabbe's disease. Hence, in such scenarios, the utilisation of broadly accessible and minimally invasive strategies, such as plasma enzymatic assay, serves as an effective and well-established approach for validating variants.^13^ In another complex clinical case (clinical vignette 2), the patient presented in the first decade of life with a pure neuropathy with conduction velocity slowing and albuminocytologic dissociation. Considering the age of onset, the slightly increased lactate level and even the albuminocytologic dissociation, a mitochondrial disease was within initial diagnostic possibilities. However, the unavailability of fast DNA-based analysis pushed us towards an immunomodulatory treatment trial, which proved inefficacious. We only then pursued genetic analysis. Molecular analysis, in this specific case, from the beginning, would avoid the need for the use of immunoglobulin, which is known to be expensive, in addition to avoiding exposing the patient to the potential risks inherent to this treatment. This is an example of how the lack of access to all investigational strategies from the beginning can influence in clinical approach, which can be potentially harmful and even less cost-effective. But again, the molecular analysis, needed to be cautiously interpreted. In this case, the two deleterious variants in POLG gene were identified in *trans* after segregation analysis in the parents. However, the association of this phenotype with POLG disorders was uncertain and given the age of the patient and doubts regarding the real impact at tissue level we pursued a muscle biopsy. The presence of COX negative fibres and the multiple deletions of mtDNA proved instrumental in reinforcing the hypothesis of a POLG-associated damage.^14^ Nevertheless, it should be said that although it does not necessarily links this gene to the neuropathy, the negative result for other causes in a comprehensive molecular analysis and the proven consequence at a tissue level favourably argued for this exceptionally atypical presentation of POLG gene.

Interpreting VUS is challenging and requires in-depth evaluation. The ACMG classification is patient-independent and primarily relies on various factors such as population frequency analysis, deleteriousness scores from different pathogenicity prediction tools, variant genomic localisation, and comparison with known pathogenic variants.^15^ Segregation analysis, whenever feasible, serves as a potent and robust tool. However, for specific variants, experimentally measuring the functional consequences of the variant becomes crucial.^15^ The third case illustrate that in which we needed an experimental Xenopus oocyte model to prove functional impact and hence pathogenicity of the variant found on KCNA1. To develop the infrastructure and to optimize the protocols in our site would be time demanding and costly, but this was achieved within the ICGNMD consortium.

Clinical vignettes four, five and six illustrate the importance of deep phenotyping to guide molecular analysis and clinical reasoning about the pathological process affecting the patient. In all cases, the clinical presentation differed from what was anticipated from known pathogenic variants in well-known genes. Although atypical presentations might be attributed to known genes, cautious clinical reasoning is necessary as a double trouble might be present. Case 4's hypermobility did not align with IGHMBP2 diagnosis. Case 5's proximal and axial weakness with myopathic MUAPS in EMG differed from expected CMT1A presentations. Case 6's abrupt clinical worsening with proximal weakness was unusual for most CMT patients. In this last case, an additional challenge is the interpretation of NCS to suggest an inflammatory neuropathy in a patient with an inherited neuropathy background. It is well known that some hereditary neuropathies might display NCS features resembling acquired conditions, as it might be the case in MPZ related disorders.^16,17^ In that sense, the clinical rapid deterioration with proximal weakness advent that really advertised for a CIDP overlapping. These cases underscore the need to continuously revisit phenotypes considering genetic diagnoses to provide optimal care. Clinicians should be aware that even with a ACMG class 5 variant, the lack of clinical guidance can lead to misdiagnosis. A comprehensive and detailed clinical assessment is crucial for accurately identifying the underlying causes.

Lastly, we highlight the limitations of molecular diagnosis and the need of interpreting it in the view of clinical thorough evaluation, combining deep and reverse phenotyping. Of note, ‘traditional’ WES analysis has some clear limitations, as it is classically known to miss copy number variation, large structural deletions, non-coding variants and DNA methylation disorders.^18^ Relying solely on WES analysis for point mutations, without exploring other tests could impede clinicians from reaching a precise diagnosis. The last three presented cases in this study serve as valuable illustrations of two significant pitfalls encountered during this diagnostic process: (i) repeat expansion disorders and (ii) Large structural abnormalities. In case 7, the comprehensive phenotyping allowed to identify a full-blown CANVAS syndrome on top of a MFN2 neuropathy. This was instrumental to guide RFC1 repeat expansion evaluation as previous WES only revealed the MFN2 point mutation. Interestingly, her brain MRI (Supplementary Fig. 9) revealed a hyperintensity in right medulla with 0.6 × 0.7 cm and no expansive effect. The image persisted without further expansion in a repeated exam. Local radiology team reported it as a probable asymmetric HOD. There was also a mild cerebellar atrophy in VI and VIIa cerebellar lobules as it has been described earlier in CANVAS.^19^ Patient had no palatal tremor, and to the best of our knowledge, HOD has not been described in neither genetic disorders and whether it might represent and additional feature of CANVAS remains a matter of future research. Cases 8 and 9 serve as examples of further analysis that can be applied to NGS data beyond solely looking for point mutations in ‘traditional’ WES script.

As we had seen in the past with NGS, the cost of third-generation sequencing techniques should decrease in the future, so it is expected that this new and advanced methods, which can detect structural abnormalities, expansions, and other complex genomic variations, will play a significant role in making important clinical decisions regarding the identification and pathogenicity of genetic findings. But as we shown here, the clinical reasoning materialized in deep and reverse phenotyping are crucial for guiding and interpreting this data.

The Table 1 summarize the cases and learning points.

**Table 1:** Summary of clinical vignettes and learning points

Gene(s)	Variant	Additional learning point
Reverse phenotyping and variant validation		
GALC (Hom)	c.973A>G	Misdiagnosis with acquired condition
POLG (Comp Het)	c.2557C>T and c.1943C>T	Misdiagnosis with acquired condition
KCNA1	c.902G>A	Collaboration for variant validation
Deep phenotyping and identification of the best molecular approach		
IGHMBP2 (Hom) and ALDH18A1 (Hom)	c.2796delC and c.121C>T	Double hit genetic disorder
PMP22 (Het) and MYOT (Het)	chr17 dup and c.179C>T	Double hit genetic disorder
MPZ (Het)	c.292C>T	Double hit genetic and acquired disorder
Exploration of genomic tests beyond whole exome sequencing (WES)		
MFN2 (Het) and RFC1 (Hom)	c.2207A>T and AAGGG RE	Double hit genetic disorder with RE
SPG11 (Hom)	Large deletion	WES hidden variant in known gene
MPZ (Het)	Large deletion	WES hidden variant in known gene

The table summarizes the nine presented clinical vignettes with its molecular diagnosis. Cases are grouped in three major pitfalls categories and additional learning points are highlighted for each case. Abbreviations: Hom, homozygous; Het, heterozygous; Comp Het, compound heterozygous; RE, repeat expansion; WES, whole exome sequencing.

## Conclusion

Through a case-based approach, we demonstrated the lessons learned from an international consortium for genomics in NMDs in a middle-income country. Collaborative efforts such as this have the potential to overcome local obstacles, strengthen scientific capabilities, foster diverse multi-ethnic cohorts, provide improved care for patients and even eligibility to variant-specific clinical trials.

As new technologies continue to advance and aid in uncovering hidden genetic disorders, the key to the best diagnostic approach lies in leveraging the full range of tools already available to us. Embracing all opportunities, including deep phenotyping and reverse phenotyping, stands at the forefront of this ongoing debate. By conducting comprehensive and detailed phenotypic assessments, we can better understand the clinical manifestations of genetic conditions and identify potential genetic variants. Simultaneously, employing reverse phenotyping, which involves starting with genetic data and correlating it with observed phenotypes, can be a valuable strategy to guide the diagnostic process. The integration of both approaches allows for a more robust and accurate diagnosis, leading to improved patient outcomes and personalized medical management.

Nevertheless, if symptoms related to other conditions, acquired, or inherited, are mistakenly grouped together, misdiagnosis will occur. This underlines that deep phenotyping does not preclude the need for a second look at the phenotype. Indeed, genotype analysis may reveal variants of uncertain significance, or it might not explain all the features encountered. In these cases, looking again to the phenotype, in a targeted way, might allow for variant validation, to expand the clinical features of a known variant or even to signal that a second explanation must be sought. Joining an international collaborative effort like ICGNMD provided us with local training in neurogenetics and access to a highly trained and specialized infrastructure. This significantly facilitated the diagnostic process and proved instrumental in optimising patient care in our practice.

## Supplementary Material

fcae342_Supplementary_Data

## Data Availability

Data sharing is not applicable to this article as no new data were created or analysed in this study.
