# Supplementary material for: Overcoming genetic neuromuscular diagnostic pitfalls in a middle-income country
Source: Brain Commun. 2024 Nov 14;6(6):fcae342. doi: 10.1093/braincomms/fcae342 (PMC11562110; doi:10.1093/braincomms/fcae342)
Supplement: fcae342_Supplementary_Data [file fcae342_supplementary_data.pdf]

Supplementary Material

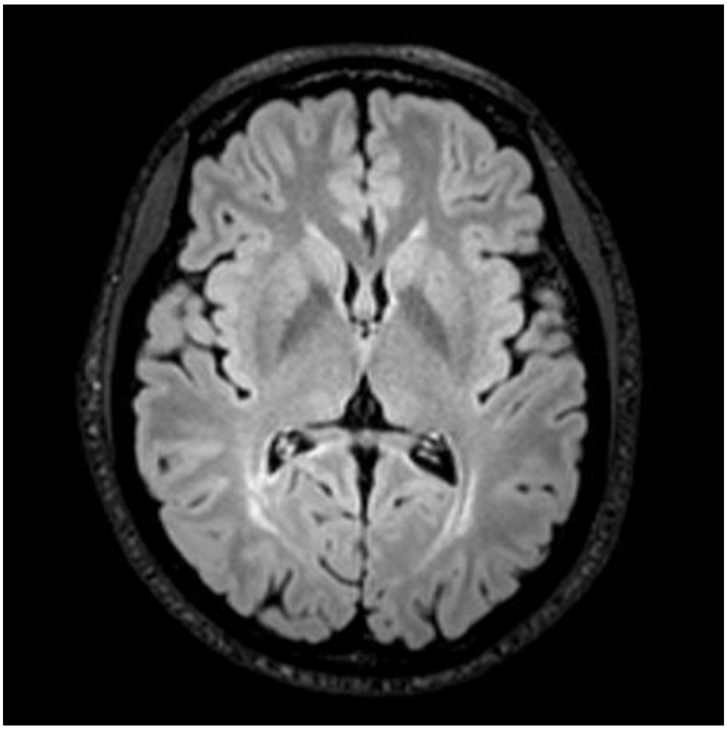

Supplementary figure 1. *Axial brain FLAIR image revealing minor white matter hyperintensities next to occipital horns of lateral ventricles.*

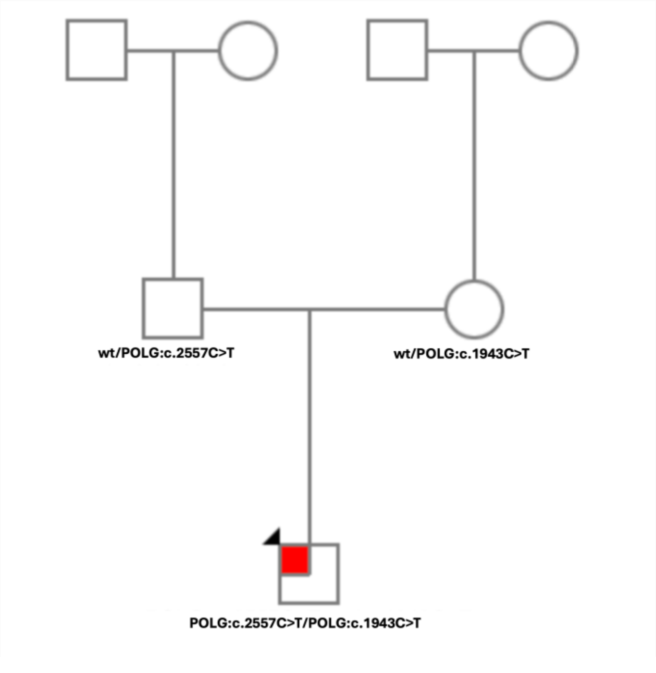

Supplementary figure 2. *Pedigree of the patient depicted in clinical vignette 2 showing the compound heterozygosity in POLG gene.*

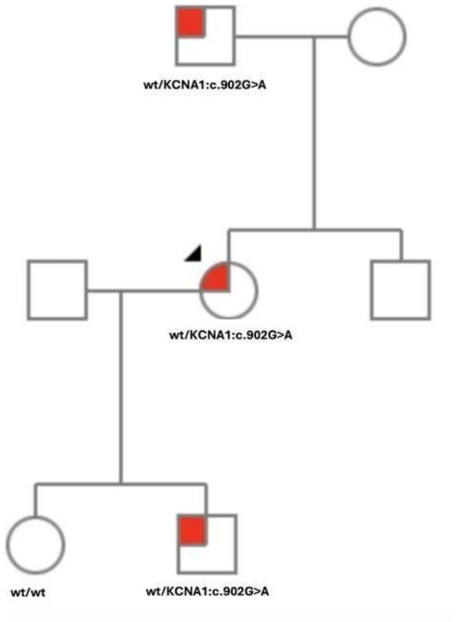

***Supplementary figure 3**. Pedigree of the patient depicted in clinical vignette 3 showing autosomal dominant pattern of inheritance.*

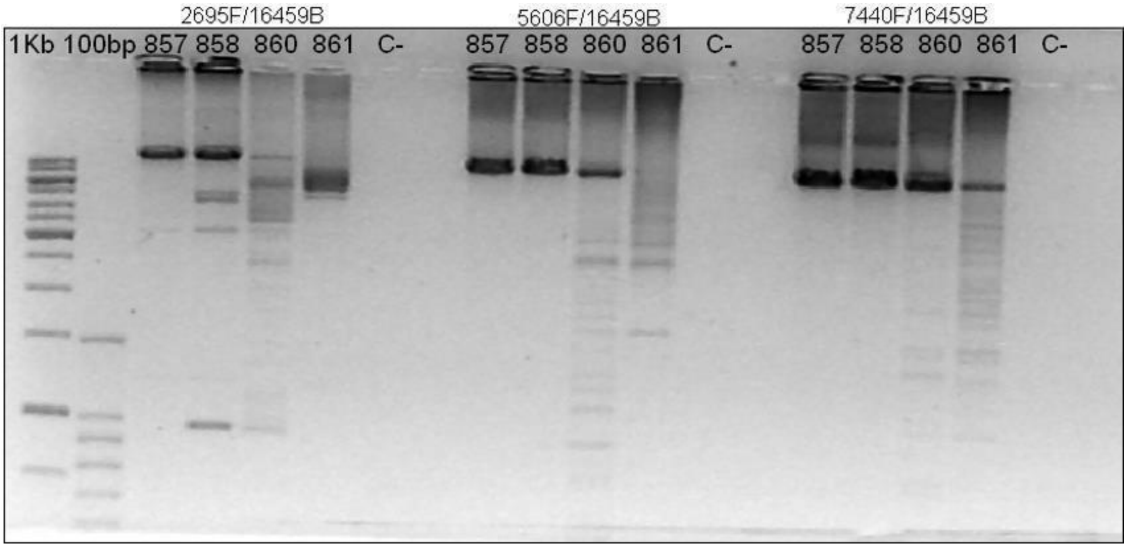

***Supplementary figure 4**. Uncropped original PCR gel from manuscript figure 3 showing different control and tested patients with specific coordinates highlighted in the top of each group of columns. The depicted patient is number 860. Number 857 is control and patients' number 858 and 861 also present mtDNA multi deletion.*

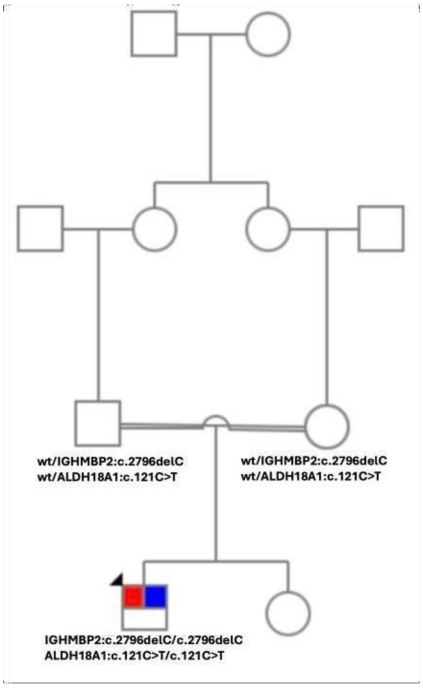

Supplementary figure 5. *Pedigree of case depicted in clinical vignette 4 revealing the consanguineous parents and the double hit homozygous disorders.*

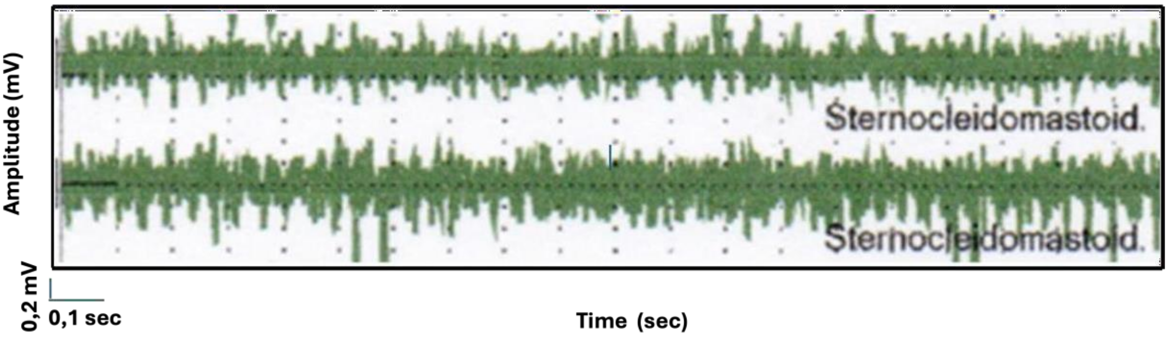

Supplementary figure 6. *Motor unit action potentials acquired through needle examination of sternocleidomastoid muscles revealing short duration, low amplitude, short recruitment, polyphasic motor unit potentials. The low amplitude (less than 2 mV) throughout the muscle contraction characterizes a "myopathic envelop "pattern, highly consistent with a myopathic process, in keeping with MYOT diagnosis on top of CMT1A.*

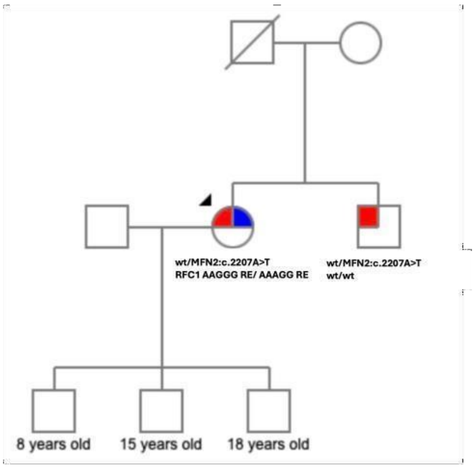

Supplementary figure 7. *Pedigree of patient depicted in clinical vignette 7 revealing the double hit genetic disorder in the proband (MFN2 and RFC1 genes) and isolated MFN2 variant in the brother. Proband's are asymptomatic, however, still young and haven't been tested for the MFN2 variant. Proband's parents were deemed asymptomatic. Father died in the 60's of unknown cause.*

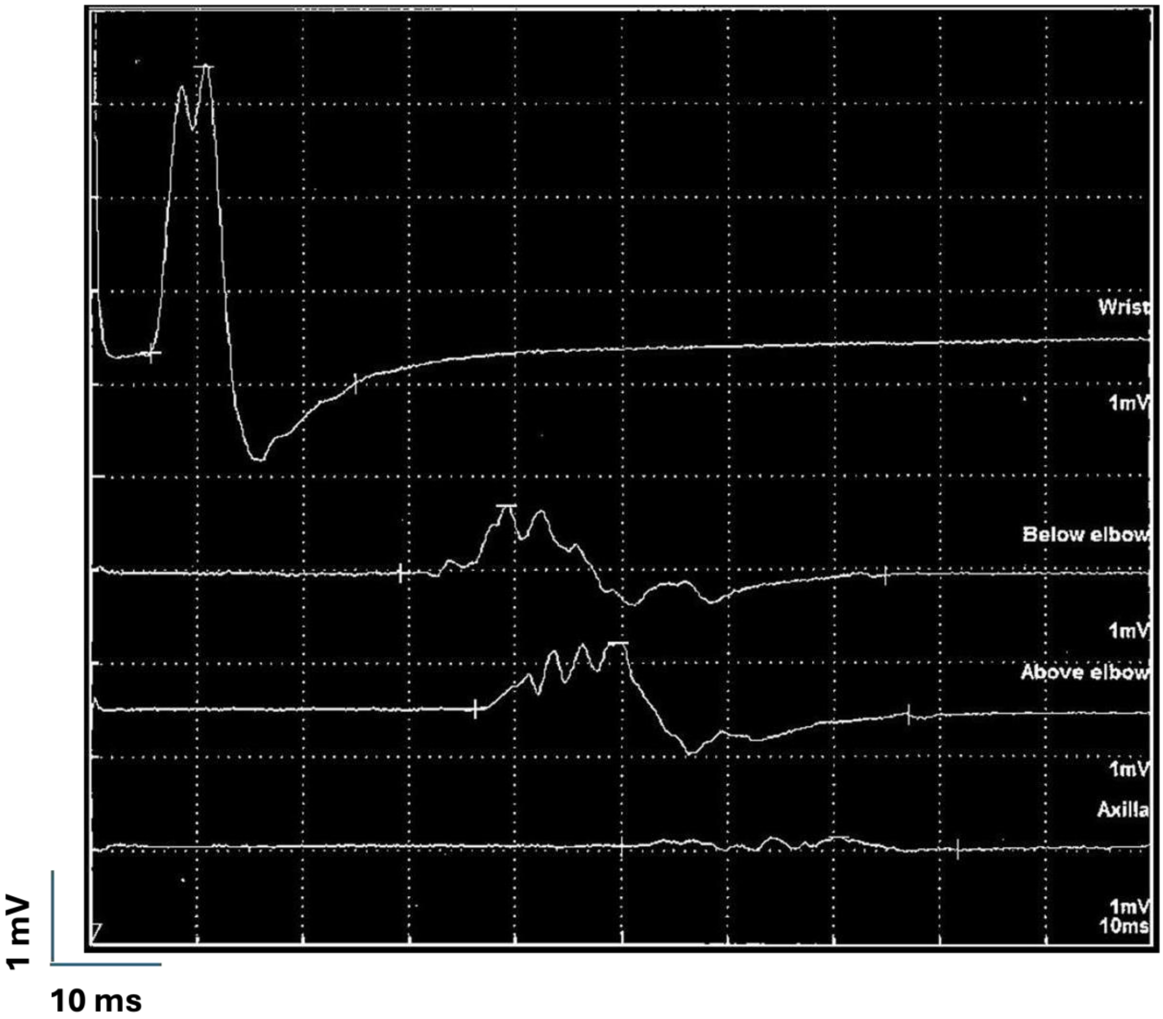

Supplementary figure 8. *Compound muscle action Potential (CMAP) of right ulnar nerve revealing visual pattern of temporal dispersion in below elbow stimulation with amplitude drop from 3.1mV to 0,8 mV (74,1% drop). Temporal dispersion exceeded 100%, thus preventing the definition of an unequivocal conduction block.*

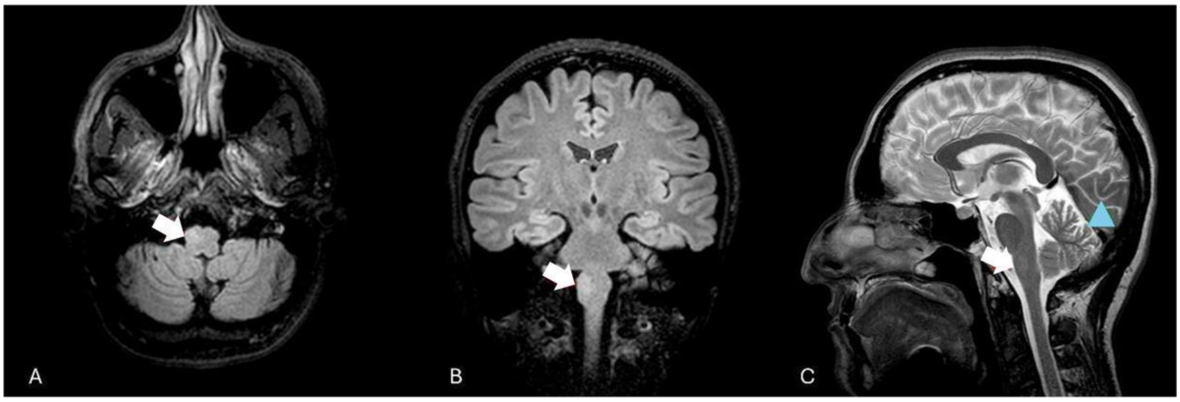

Supplementary figure 9. *In "A", axial FLAIR brain image reveals asymmetric hyperintensity in right medulla, highlighted in white arrow. In "B", a FLAIR coronal brain image and in "C", a sagittal FLAIR brain image reveals the same lesion with same white arrow pointing. The blue arrowhead in "C" points to mild cerebellar atrophy with predilection for VI and VIIa lobules, as has been described before in CANVAS patients* [1]

Reference presented in Supplementary Material

1. D.J. Szmulewicz, J.A. Waterston and G.M. Halmagyi, Sensory neuropathy as part of the cerebellar ataxia neuropathy vestibular areflexia syndrome, Neurology 76(22) (2011), 1903–1910.
